# Research on water environmental indicators prediction method based on EEMD decomposition with CNN-BiLSTM

**DOI:** 10.1038/s41598-024-51936-5

**Published:** 2024-01-19

**Authors:** Zhaohua Wang, Longzhen Duan, Dongsheng Shuai, Taorong Qiu

**Affiliations:** 1 School of Mathematics and Computer Sciences, Nanchang University, Nanchang, China; 2 Jiangxi Zhonggan Investment Survey and Design Limited Company, Nanchang, China

**Keywords:** Environmental impact, Environmental sciences

## Abstract

Water resources protection is related to the development of the social economy, and the monitoring and prediction of water environmental indicators have important practical significance. In view of the seasonality, periodicity, uncertainty, and nonlinear characteristics of water quality indicators data, traditional prediction models have poor performance. To address this issue, this paper introduces a hybrid water quality index prediction model based on Ensemble Empirical Mode Decomposition (EEMD), combined with Convolutional Neural Network (CNN) and Bidirectional Long Short-Term Memory Network (BiLSTM). We have conducted out experiments to predict dissolved oxygen based on the water quality monitoring indicators of the Liaohe National Control Sanhongcun Village station in Yichun City. The results show that the model proposed in this paper improves the $R^2$ index by 5%, 7% and 5% compared to the suboptimal model in the 4-h, 1-day and 2-day index predictions, respectively.

## Introduction

In recent years, with the development of socio-economy, water pollution has garnered escalating public attention, leading to water resource protection being widely recognized as a societal consensus. The dynamic monitoring of changes in water quality, coupled with the implementation of water environment indicator predictions, holds profound practical significance for the preservation of water resources.

The prediction of water environment indicators involves the identification of temporal changes in water quality indicators and their correlation with hydrological, meteorological, and other factors within a specified spatiotemporal context^1^. Water environment indicator prediction can be categorized into mechanistic prediction methods and non-mechanistic prediction methods, depending on their underlying theoretical foundations.

Mechanistic prediction methods are holistic approaches grounded in the governing principles and evolving dynamics of the water environment, encompassing diverse disciplines such as hydrodynamics, ecology, and chemistry^2^. These methods typically employ models to encapsulate the intricate interplay among various elements. Commonly utilized models in this category include the Water Quality Analysis Simulation Program (WASP)^3^, QUAL model^4^, MIKE system^5^, Generalized Watershed Loading Function (GWLF)^6^, and others.

In contrast, non-mechanistic prediction methods adopt a ’black box’ approach. These models rely on probabilistic statistical theories and are tailored to specific water environments, demonstrating effective predictive capabilities. Three prevalent non-mechanistic models can be identified: traditional probabilistic statistical models, such as the grey model^7^ and Markov chain model^8^; time series models, such as Exponential Smoothing (ETS) and Auto Regressive Integrated Moving Average (ARIMA); and artificial intelligence models, including Support Vector Machine (SVM), Long Short-Term Memory (LSTM), eXtreme Gradient Boosting (XGBoost), Gate Recurrent Unit (GRU), and Informer, among others.

Surface water is an important type of water environment. Its water quality indicators exhibit characteristics such as seasonality, periodicity, uncertainty, and nonlinearity. There are also complex dependent relationships between the indicators^9^. Traditional probabilistic statistical methods are difficult to model such complex dependent relationships. At present, artificial intelligence methods represented by deep learning have made great progress in the application of surface water environment indicator prediction. Recurrent neural networks (RNNs) are suitable for processing time series data, but they suffer from the problem of gradient disappearance. To solve the problems in RNNs, Hochreiter et al.^10^ proposed LSTM networks,which can perform long time series prediction tasks. Hu et al.^11^ used LSTM to predict pH and water temperature in water quality indicators, and Zhang Yiting et al.^12^ applied LSTM to the prediction of ammonia nitrogen indicators in river water quality. However, a single LSTM model cannot avoid the interference of noise, resulting in unsatisfactory prediction accuracy. To solve the noise interference problem, convolutional neural networks (CNNs) are introduced to extract features from multidimensional time series, such as: Zhang Mingwei et al.^13^ employed the CNN-LSTM model to predict the dissolved oxygen index of river water quality, and Wang Zhibo et al.^14^ employed CNN-LSTM to predict the dissolved oxygen index of lake water quality. But LSTM can only make predictions based on historical data, while water quality indicators are not only related to historical data, but also related to future data. On the other hand, modal decomposition methods are introduced to eliminate the impact of noise, such as: Yuan Meixue et al.^15^ employed wavelet decomposition to denoise water quality data, and then used a hybrid LSTM and Seq2Seq model for prediction. Benjamin et al.^16^ applied the Empirical Mode Decomposition (EMD) method to decompose the dissolved oxygen indicator in the water quality time series, effectively isolating the trend and fluctuation components of the data. José et al.^17^ employed EMD and LSTM to improve the performance of time series classification. Bai Wenrui et al.^18^ first employed Variational Mode Decomposition(VMD) to decompose water quality indicators, and then used LSTM to predict water quality indicators.Wavelet decomposition has defects such as edge effects and difficulty in determining the basis function; while VMD requires higher data stability and linearity.

This paper proposes a CNN-BiLSTM water quality indicator prediction model based on Ensemble Empirical Mode Decomposition (EEMD) decomposition, aiming to overcome the prevalent challenges in deep learning applications for water quality indicator prediction, as well as to address the periodicity, uncertainty, and nonlinearity inherent in water quality monitoring data. EEMD effectively mitigates the issue of mode mixing encountered in EMD and imposes less stringent requirements on data stationarity and linearity compared to VMD. CNN is employed to extract local features from water quality indicator data, while BiLSTM handles sequential dependence modeling within this data, considering the impacts of both forward and backward data. To validate the efficacy of our proposed model, we conducted multivariate and multi-step prediction experiments using water quality data obtained from the national monitoring station in Sanhong Village, Liaohe.

## Model and methods

### Water environment indicator decomposition

EEMD was proposed by Wu et al.^19^based on Empirical Mode Decomposition (EMD) to overcome the problem of mode mixing in EMD decomposition.

EEMD is a method that involves adding Gaussian white noise to the original sequence, applying EMD to the sequence multiple times according to a predefined number of experiments, and then taking the average of the decomposition results to eliminate the influence of noise. This methodology imparts properties of uniform distribution and smoothness to the original sequence.The steps for sequence decomposition in EEMD are as follows:

(i) Add white noise of limited amplitude to the original indicator sequence to obtain a new sequence:
  1 $$\begin{aligned} X^s=X+\varepsilon ^s \end{aligned}$$
  where $X(X \in R^{(m \times n)})$ is the original sequence, $\varepsilon ^s$ is white noise,and $X^s$ is the new sequence.
(ii) Decompose $X^s$ into Intrinsic Mode Function(IMF) components using EMD:
  2 $$\begin{aligned} X^s=\sum _{l}^{L}C_l^{EMD,s} +r(t) \end{aligned}$$
  where $C_l^{EMD,s}$ is the intrinsic mode function after EEMD decomposition, *r*(*t*) is residual.
(iii) Repeat the above steps according to the set number of times and calculate the final result:
  3 $$\begin{aligned} X=(C_1^{EMD, s}, C_2^{EMD,s},..., C_L^{EMD, s}, r(t)) \end{aligned}$$

**Figure 1:**
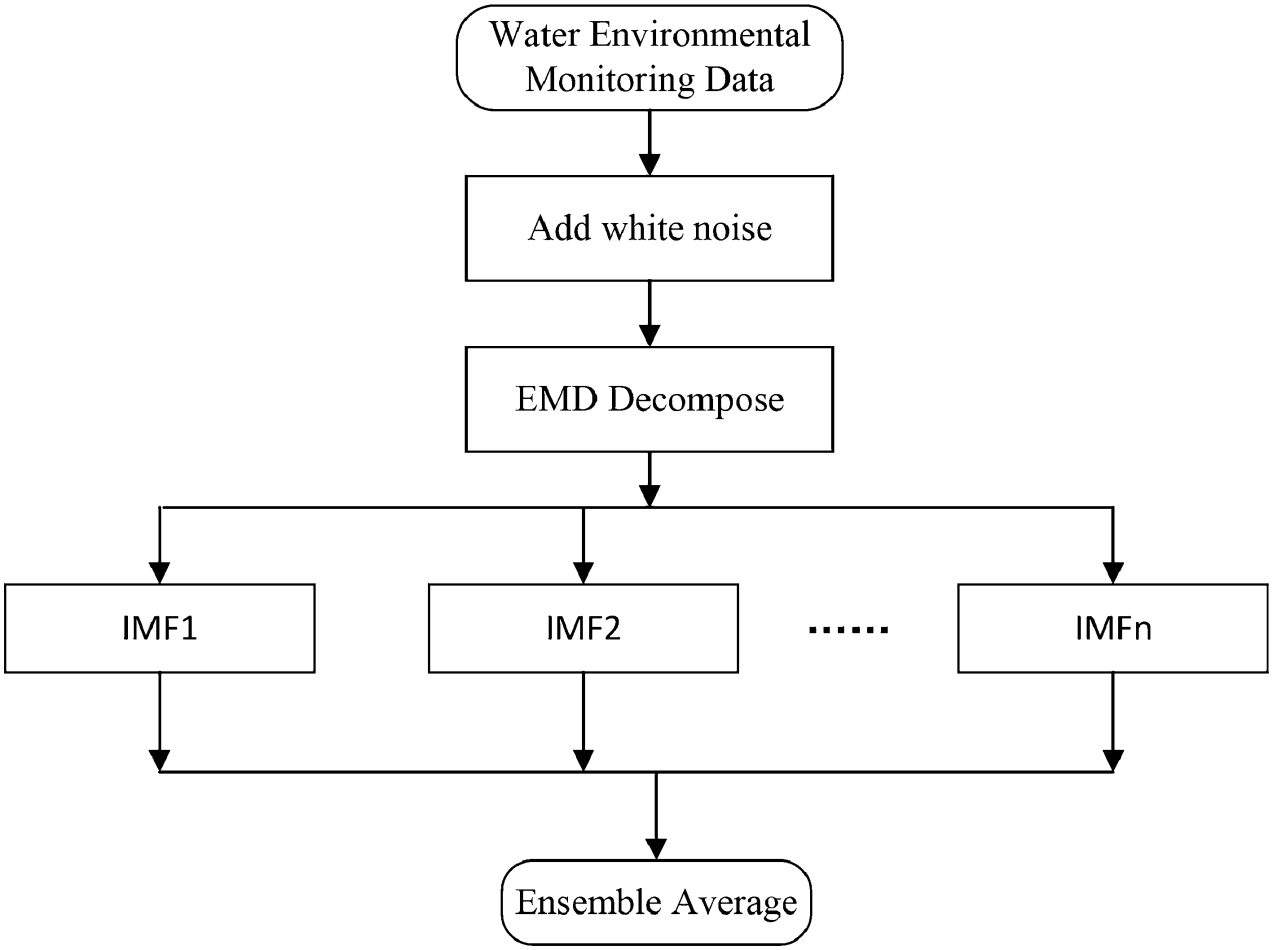
EEMD decomposition flowchart.

The process flow of EEMD decomposition for water quality indicators is illustrated in the Fig. 1.

### Local correlation feature extraction of water environment indicators

Convolutional neural networks (CNN) are feedforward neural networks that use convolution and pooling operations for feature extraction. It is an important algorithm in deep learning. For time series data, 1D convolutions are often used.

In this paper, a sliding window is employed on the water environment indicator sequence to extract local features. Additional noise filtering is carried out through convolution and pooling operations to achieve enhanced outcomes. The specific formula is as follows:

4 $$\begin{aligned} Y = w*X \end{aligned}$$

where *w* is the convolution kernel, $*$ denotes convolution, *X* represents the water quality indicator sequence that has been decomposed by EEMD, and *Y* is the extracted feature.

### Temporal dependence modeling of water environment indicators

This paper chooses BiLSTM to model temporal dependencies. BiLSTM constitutes an advancement over the LSTM neural network. Relevant research^20^ indicates that BiLSTM offers noteworthy enhancements in performance compared to LSTM for time series prediction tasks.

5 $$\begin{aligned} H=BiLSTM(Y) \end{aligned}$$

where *Y* represents the vector of target variables to be predicted, *H* represents the prediction results. BiLSTM consists of two layers of LSTM neural networks that operate in opposing directions.Rather than merely stacking the two LSTM layers, it integrates data features from both forward and backward directions at the present time step for predictive purposes.

### Model building

Given the strong coupling and nonlinear characteristics of water environment monitoring data, traditional prediction methods often yield subpar results.Accordingly, this paper introduces a CNN-BiLSTM hybrid model for water environment data prediction based on EEMD decomposition.

Initially, the preprocessed water environment data is decomposed by EEMD, yielding four modes. Each of these modes is subsequently fed into both CNN and BiLSTM for feature extraction. Ultimately, the extracted features are accumulated and reconstructed to derive the predictive outcome.

**Figure 2:**
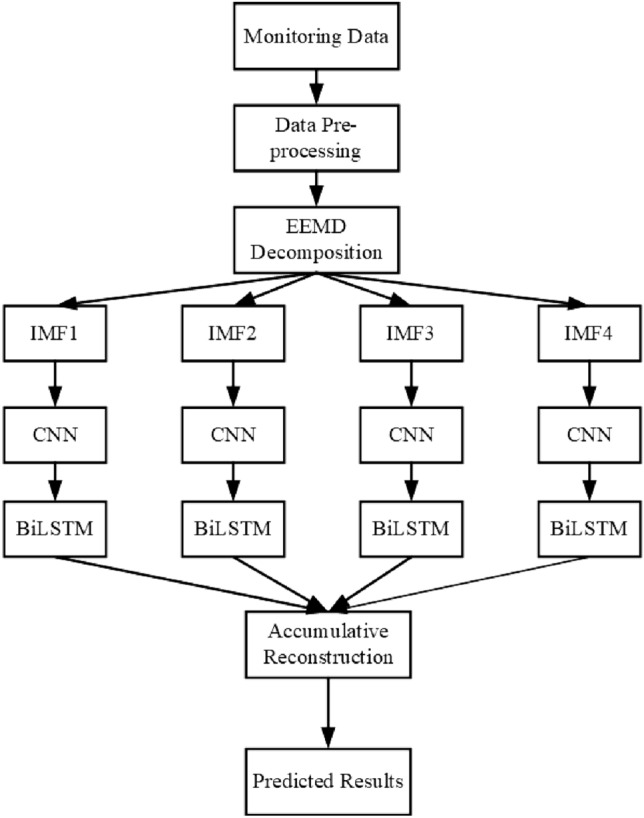
EEMD-CNN-BiLSTM Mixture model Diagram.

This hybrid model synergistically integrates EEMD, CNN, and BiLSTM to capitalize on the strengths of each component: EEMD for noise reduction, CNN for capturing local features, and BiLSTM for modeling sequential dependencies. The ensemble methodology has the potential to enhance prediction accuracy. In this experiment, dissolved oxygen is decomposed by EEMD, and then combined with other indicators to form new training data. The model structure is illustrated in the Fig. 2.

## Experiments

### Dataset

The research focuses on water quality monitoring data obtained from the national monitoring station in Sanhong Village, Liaohe.Liaohe is the largest tributary of Xiuhe River,which traverses Jing’an County in Yichun City. It holds significance as the primary river in the county and eventually merges into Poyang Lake via the Xiuhe River.

The monitoring dataset spans from November 2020 to December 2022,with measurements taken every four hours, amounting to a total of 4,700 data points. It encompasses nine indicators: water temperature (TEMP),pH,dissolved oxygen (DO),potassium permanganate (PP),ammonia nitrogen (TAN),total phosphorus (TP),total nitrogen (TN),electrical conductivity (EC),and turbidity (TUB).This dataset is obtained from the Environmental Quality Information Release Platform of Jiangxi Province.

In addition, meteorological data from Yichun City covering the same time period was also gathered, encompassing six indicators:temperature,atmospheric pressure,humidity,wind speed,dew point temperature,and precipitation.This data is obtained from the website “Reliable Prognosis”.

Among the various water quality indicators, the concentration of dissolved oxygen serves as a crucial benchmark for assessing water quality^21^. Consequently, this paper focuses on utilizing dissolved oxygen as the target indicator for model prediction.

**Figure 3:**
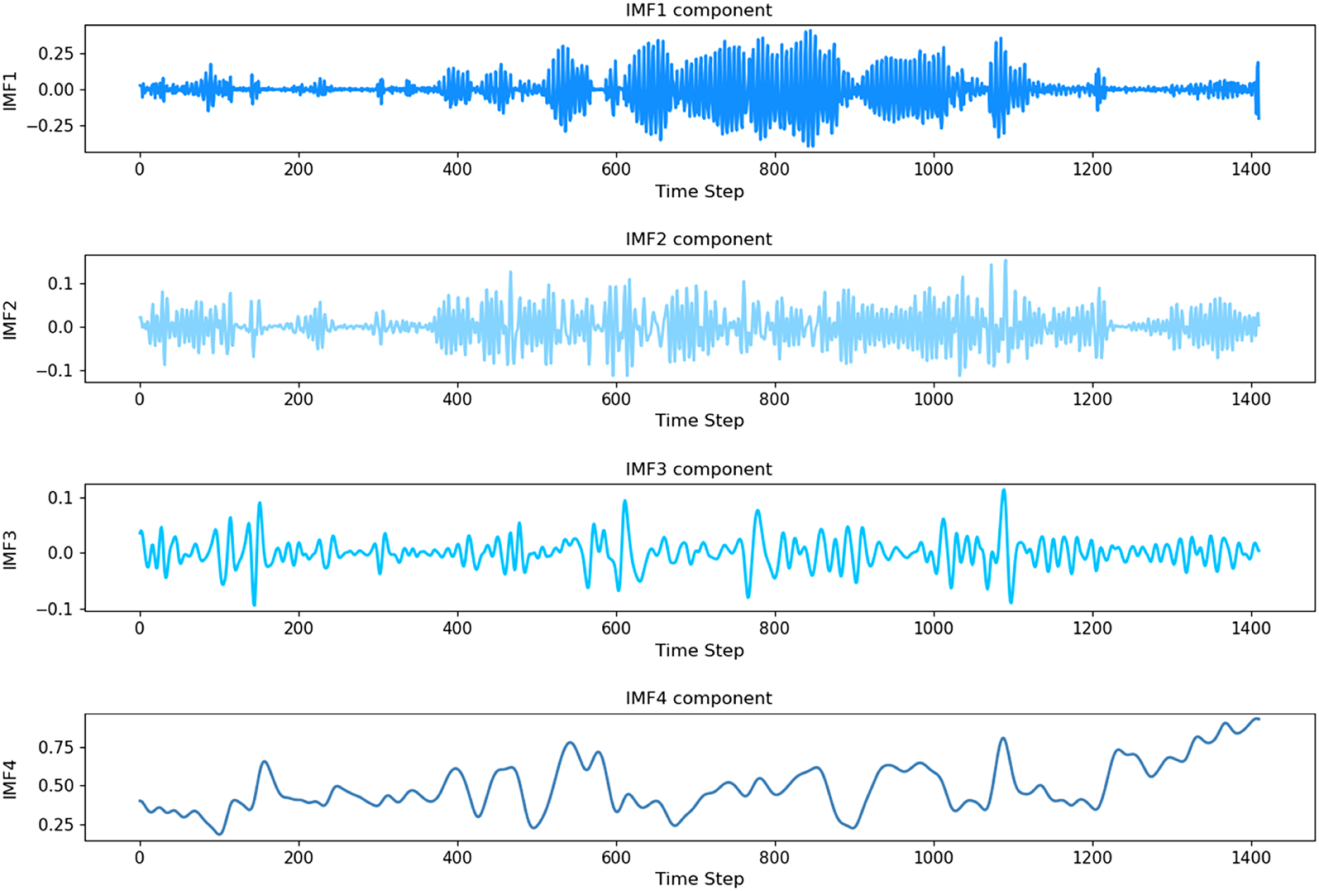
Dissolved oxygen index after decomposition of EEMD.

Through a series of experiments and evaluations, it was determined that ’4’ was the optimal number of modalities, as it demonstrated the best performance and accuracy during model training. In this paper, the EEMD method (4 modes) is employed to decompose the dissolved oxygen indicator through experimental comparison. The waveform diagrams of each mode after decomposition in the validation and test sets are illustrated in Fig. 3:

Through autocorrelation experiments, we observed that the three modes: IMF1, IMF2, and IMF3 exhibit evident cyclical characteristics, while IMF4 retains the trend characteristic inherent in the data.

(i) Missing and outlier value handling

During the analysis of the data, it was discovered that certain issues such as missing values and outliers existed due to factors like equipment maintenance or malfunctions that occurred during the data collection process.

For indicators with a significant number of consecutive missing values, linear interpolation is employed to fill in the gaps according to the formula:

6 $$\begin{aligned} \varphi \left( x \right) = \frac{x-x_{1} }{x_{0}-x_{1}} y_{0} +\frac{x-x_{0} }{x_{1}-x_{0}} y_{1} \end{aligned}$$

where x represents time, $\varphi \left( x \right)$ represents the estimated value at that specific time x. The coordinates $x_{0}$ and $y_{0}$ represent the first known data point, $x_{1}$ and $y_{1}$ represent the second known data point.

(ii)Normalization

As water quality indicators possess distinct scales, for optimal model training, each indicator is normalized using the formula:

7 $$\begin{aligned} x^{'} =\frac{x-min(x)}{max(x)-min(x)} \end{aligned}$$

where x is the original data that needs to be normalized, $x^{'}$ is the normalized data, and its value range is [0,1], *max*(*x*) and *min*(*x*) are the maximum and minimum values in the dataset, respectively.

(iii) Correlation analysis

**Figure 4:**
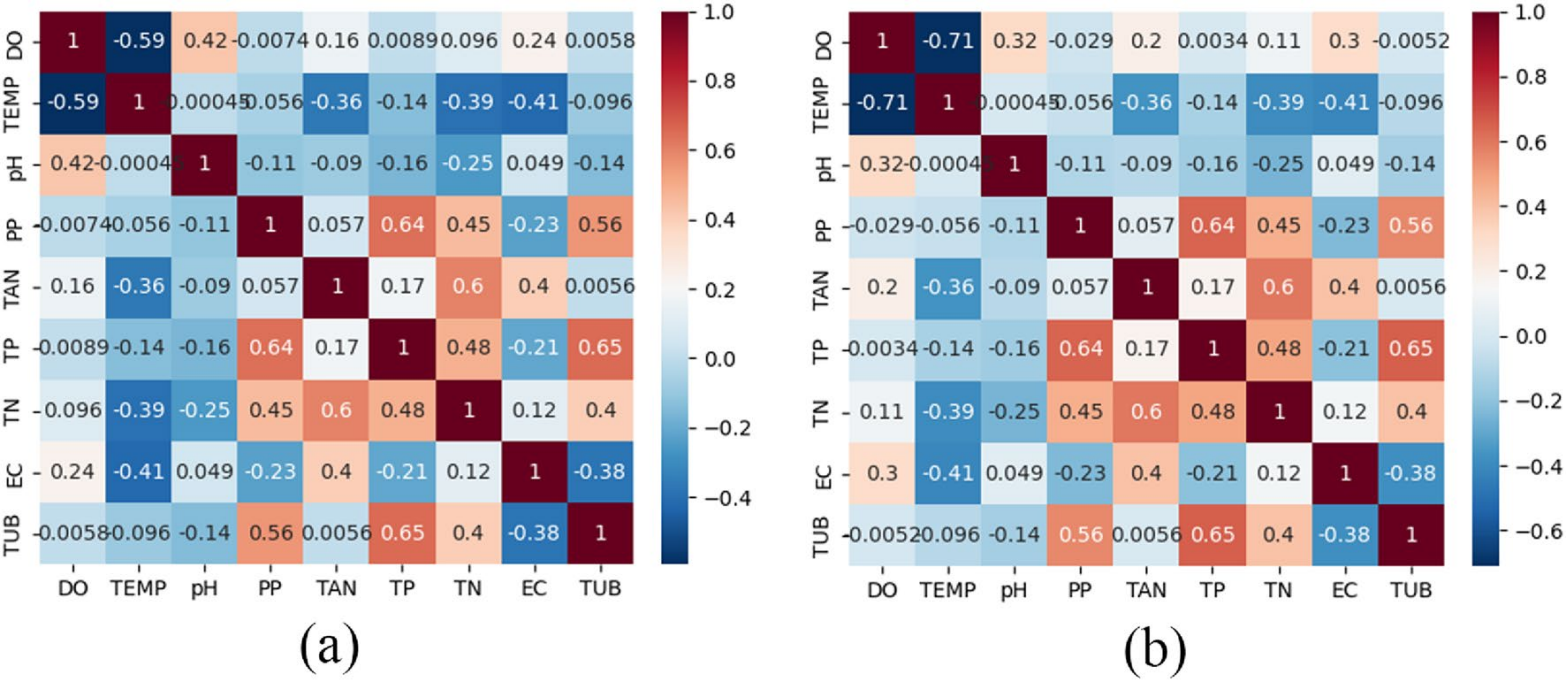
Heat map: (**a**) is correlation between water quality indicators, (**b**) is IMF4 correlation heat map after EEMD decomposition.

To investigate the significance of each indicator in the prediction process, correlation analysis is conducted on the data, and a correlation heat map is presented in the figure 4.

It is evident that following EEMD decomposition, the correlations between dissolved oxygen and various indicators such as temperature, electrical conductivity, ammonia nitrogen, and total nitrogen have demonstrated an increase.

### Determination of model parameters

In this paper, grid search is employed to optimize the model parameters. Only one parameter is adjusted at a time, and grid search is utilized for fine-tuning. Through iterative execution of the aforementioned steps, the optimized model parameters are presented in Table 1:

**Table 1:** Model parameters for each model.

Indicators	XGboost	LSTM	GRU	BiLSTM	CNN-BiLSTM	Informer	Ours
Training set	0.7	0.7	0.7	0.7	0.7	0.7	0.7
Validation set	0.15	0.15	0.15	0.15	0.15	0.15	0.15
Test set	0.15	0.15	0.15	0.15	0.15	0.15	0.15
Batch size	–	256	256	256	512	256	256
Input window	8	8	8	8	8	8	8
Loss function	–	MSE	MSE	MSE	MSE	MSE	MSE
Learning rate	0.1	0.001	0.001	0.001	0.001	0.001	0.001
Weight decay	–	0.0001	0.0001	0.0001	0.0001	0.0001	0.0001
Stacking depth	–	1	1	1	1	–	1
Hidden layer dimensions	–	256	256	32	256	–	256
Training epochs	100	300	300	300	300	300	300
CNN Output channels	–	–	–	–	128	–	128
CNN Convolution kernel size	–	–	–	–	–	–	1
CNN Convolution stride	–	–	–	–	–	–	1

Since each model has different characteristics,the parameters that need to be set are not exactly the same.In the table, “–” indicates that the model does not need to set this parameter.In order to facilitate the comparison of model performance, the same parameters should be set as much as possible

### Metrics for experimental evaluation

Mean absolute error (MAE),mean square error (MSE),Mean Absolute Percentage Error (MAPE) and correlation coefficient $(R^2)$ are employed as quantitative metrics to assess the predictive performance of the model.

8 $$\begin{aligned} MAE= & {} \frac{\sum \left| y- {\hat{y}} \right| }{n} \end{aligned}$$

9 $$\begin{aligned} MSE= & {} \frac{\sum (y-{\hat{y}} )^{2}}{n} \end{aligned}$$

10 $$\begin{aligned} MAPE= & {} \frac{100\%}{n} \sum \left| \frac{{\hat{y}}-y }{y} \right| \end{aligned}$$

11 $$\begin{aligned} R^{2}= & {} \frac{\sum ({\hat{y}}-{\bar{y}})^{2} }{\sum (y-{\bar{y}})^{2}} \end{aligned}$$

where *y* is the true value, ${\hat{y}}$ is the predicted value, and ${\bar{y}}$ is the mean of the indicator. When comparing models, a lower value of MAE, MSE, and MAPE indicates better model performance, while an $R^2$ value closer to 1 signifies a superior model.

### Experimental design

Dissolved oxygen is chosen as the target variable for prediction, and both single-step and multi-step predictions are carried out. Based on data correlation analysis, the following four combinations of data have been designed as described in Table 2:

**Table 2:** Combination of experimental data.

No.	Variable	Prediction target
Combination 1	TEMP, pH, PP, TAN, TP, TN, EC, TUB	DO
Combination 2	TEMP, pH, PP, TAN, TP, TN, EC, TUB + Meteorological information	DO
Combination 3	TEMP, pH, EC, TAN	DO
Combination 4	TEMP, pH, EC, TAN + Meteorological information	DO

Combination 1 employs the remaining 8 water quality indicators, excluding dissolved oxygen, as input variables. Combination 2 incorporates meteorological data into Combination 1 to assess its influence on the prediction. Combination 3 utilizes the top 4 most strongly correlated indicators as input variables. Combination 4 introduces meteorological data into Combination 3

Based on the above 4 data combinations,the experiments are designed as follows:

(i) Window size experiment:Verify the impact of window size on results.
(ii) Model comparison:Compare with mainstream time series prediction models XGBoost, LSTM, GRU, Informer.
(iii) Correlation experiment:Conduct multi-step comparative prediction experiments on four data combinations.
(iv) Ablation experiment:Verify the role of each module through ablation experiment.

### Experimental results and analysis

In this paper, relevant experiments are conducted in accordance with the aforementioned plan.

(i) Sliding Window Size Experiment: To determine the optimal window size, comparative experiments are performed using window sizes of 8 and 48 for XGBoost, LSTM, GRU, and our proposed model.

Based on the experimental results, it appears that each model demonstrates a low sensitivity to the window size.Taking the $R^2$ metric as an example,in the XGBoost model, there is only a 2% improvement in prediction results when the window size was increased to 48. However, better prediction results were observed in the other models when the window size was set to 8. Consequently, this paper opts for a window size of 8 in subsequent experiments.

(ii) Popular prediction models commonly used in the field of time series forecasting, namely XGBoost, LSTM, and GRU, are selected for comparison. In the realm of time series forecasting, several popular prediction models are commonly employed for comparative analysis. These models include XGBoost, LSTM, and GRU. In light of the widespread adoption of transformer-based models for time series prediction, Temporal Fusion Transformer (TFT) was introduced by Bryan et al.^22^ TFT is capable of learning intricate relationships between different temporal scales within time series data. Building upon this, Jitha et al.^23^ leveraged the temporal fusion transformer architecture to model and predict river water quality indicators.

Additionally, Zhou et al.^24^ proposed the Informer model for long-term time series prediction. Therefore, we conducted experiments incorporating the Informer model into our comparative analysis.

The comparison experiment is conducted at step sizes of 1 (4 hours), 6 (1 day), 12 (2 days), and 18 (3 days). The results are presented in Table 3, with the optimal results are in bold.

**Table 3:** Experiment results of model multi-step comparison.

Method	Metric	Combination 1				Combination 2			
		@1	@6	@12	@18	@1	@6	@12	@18
XGBoost	MSE	0.5690	0.8248	1.1159	1.2988	0.5863	0.7754	1.0562	1.2617
	MAE	0.5380	0.6502	0.7675	0.8400	0.5291	0.6298	0.7500	0.8317
	MAPE	0.0641	0.0778	0.0917	0.1012	0.0626	0.0756	0.0904	0.1002
	$R^2$	0.8597	0.7954	0.7217	0.6746	0.8554	0.8077	0.7366	0.6839
LSTM	MSE	0.4278	0.6828	1.0316	1.2628	0.4398	0.6940	1.0076	**1.2596**
	MAE	0.4528	0.6043	0.7354	**0.8071**	0.4682	0.5841	0.7122	**0.8080**
	MAPE	0.0539	0.0726	0.0860	**0.0935**	0.0556	0.0684	0.0826	**0.0928**
	$R^2$	0.8958	0.8325	0.7451	0.6861	0.8928	0.8298	0.7512	0.6870
GRU	MSE	0.4325	0.7187	1.0491	1.2644	0.3789	0.7087	1.0579	1.3190
	MAE	0.4498	0.6066	0.7311	0.8303	0.4221	0.5995	0.7324	0.8275
	MAPE	0.0536	0.0722	0.0849	0.0985	0.0505	0.0706	0.0842	0.0953
	$R^2$	0.8946	0.8237	0.7408	0.6857	0.9077	0.8261	0.7387	0.6721
Informer	MSE	0.4593	0.8761	1.2524	1.3496	0.3337	0.6643	1.3052	1.3650
	MAE	0.5354	0.7435	0.8839	0.9179	0.4260	0.6286	0.9033	0.9097
	MAPE	0.0745	0.0826	0.0921	0.1051	0.0654	0.0859	0.0962	0.1324
	$R^2$	0.4966	0.6624	0.7363	**0.7142**	0.3945	0.4996	0.7258	**0.7622**
Ours	MSE	**0.2306**	**0.4521**	**0.8685**	**1.1987**	**0.2037**	**0.5051**	**0.7978**	1.2857
	MAE	**0.3477**	**0.4921**	**0.6917**	0.8192	**0.3389**	**0.5011**	**0.6216**	0.8150
	MAPE	**0.0417**	**0.0619**	**0.0845**	0.1021	**0.0406**	**0.0583**	**0.0721**	0.0947
	$R^2$	**0.9438**	**0.8892**	**0.7859**	0.7028	**0.9504**	**0.8763**	**0.8034**	0.6809

According to the results, the proposed model in this paper consistently achieves the best prediction performance at step 1, 6 and 12 in Combination 1, with improvements in $R^2$ of 5%, 7%, 5% compared to the second-best model. And in step 18, the model achieved a second-best result, with a difference of only 0.01 from the optimal value. When meteorological data is introduced (Combination 2), there is a little enhancement in prediction performance observed for any of the models, and the $R^2$ values remain relatively consistent across different step sizes. Notably, the proposed model continues to deliver optimal results at step sizes of 1, 6, and 12. At the step 18,Informer performed slightly better than our proposed model, proving the advantage of the informer in long-term prediction.

As the prediction step size increases, the forecasting performance of various models tends to decline. However, the proposed model consistently achieves the best results across nearly all step sizes, demonstrating its efficacy in dissolved oxygen prediction.

**Figure 5:**
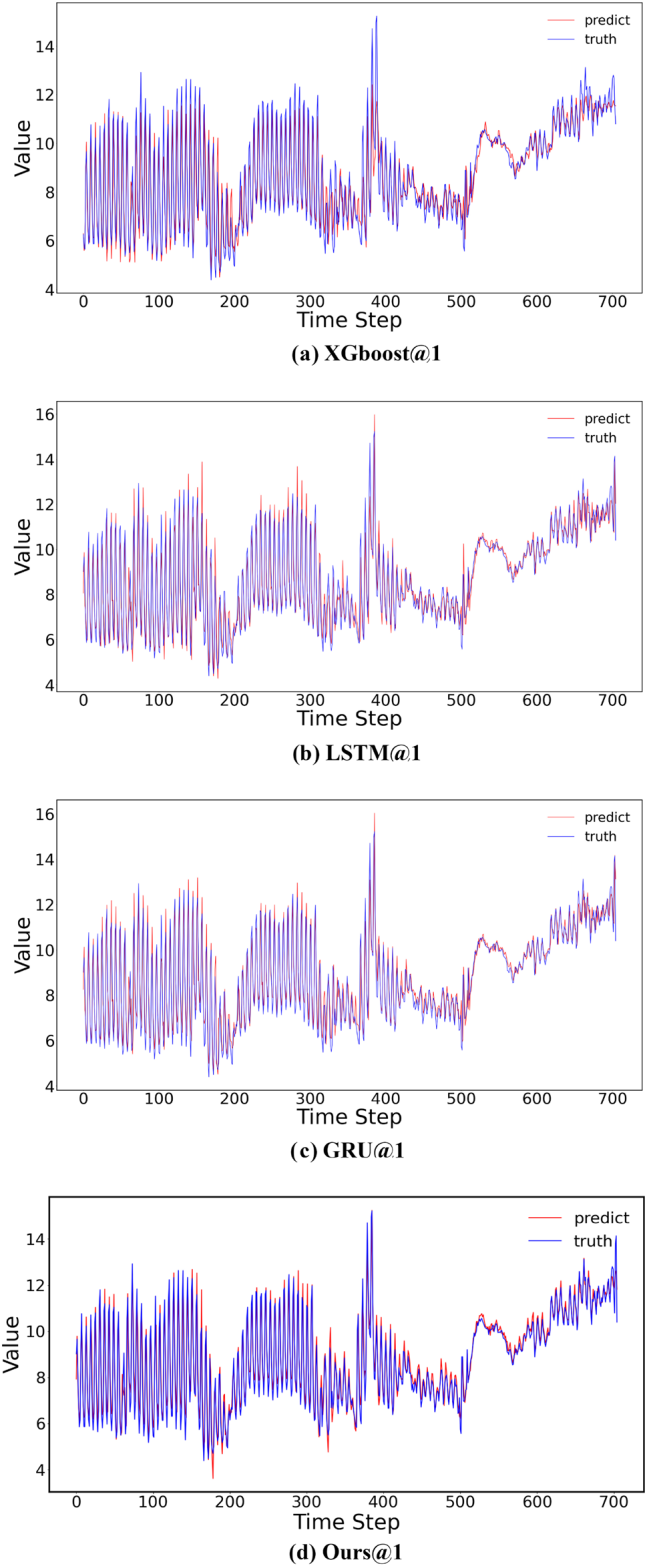
Comparison of predicting curves.

Examining the 1-step prediction curve, it is evident that the proposed model in this paper provides a better fit to the actual values, with the curves nearly overlapping the true values. The curves are depicted in Fig. 5.

(iii) Following correlation analysis, the top 4 most strongly correlated indicators are selected and utilized in conjunction with the proposed model for multi-step prediction. The results are presented in Table 4, with the optimal value are in bold for reference.

**Table 4:** Experiment results of correlation analysis.

Combination	Metric	@1	@6	@12	@18
Combination 1	MSE	0.2306	0.4521	0.8685	**1.1987**
	MAE	0.3477	0.4921	0.6917	0.8192
	MAPE	0.0417	0.0619	0.0845	0.1021
	$R^2$	0.9438	0.8892	0.7859	**0.7028**
Combination 2	MSE	**0.2037**	0.5051	**0.7978**	1.2857
	MAE	0.3389	0.5011	**0.6216**	0.8150
	MAPE	**0.0406**	**0.0583**	**0.0721**	0.0947
	$R^2$	**0.9504**	0.8763	**0.8034**	0.6809
Combination 3	MSE	0.2224	0.4738	0.9565	1.5275
	MAE	**0.3349**	0.4931	0.7060	0.8699
	MAPE	0.0414	0.0606	0.0859	0.1037
	$R^2$	0.9458	0.8839	0.7642	0.6217
Combination 4	MSE	0.3048	**0.4473**	0.8466	1.2323
	MAE	0.3956	**0.4859**	0.6535	**0.7924**
	MAPE	0.0481	0.0597	0.0780	**0.0909**
	$R^2$	0.9257	**0.8904**	0.7912	0.6944

It is evident that the prediction accuracy remains relatively consistent even after indicator screening based on correlation analysis. Specifically, Combination 3 achieves the second-best $R^2$ value in 1-step prediction, while Combination 4 attains the optimal $R^2$ value in 6-step prediction.

In summary, the selection of indicators that are highly correlated with the target allows for a reduction in data dimensionality without significantly compromising the model’s performance. The proposed model, when incorporated with these correlated indicators, continues to deliver robust multi-step dissolved oxygen forecasting. This approach enables more efficient water quality modeling by utilizing fewer but informative variables, thereby streamlining the modeling process.

(iv) Ablation Experiment: To further substantiate the contributions of individual modules within the proposed model, corresponding ablation experiments have been devised. The results are presented in Table 5, with the optimal value highlighted by bold for clarity.

**Table 5:** Experiment results of ablation experiments.

Method	Metric	Combination 1				Combination2			
		@1	@6	@12	@18	@1	@6	@12	@18
BiLSTM	MSE	0.4275	0.6730	1.0254	1.2852	0.4425	0.7167	1.0086	1.2935
	MAE	0.4538	0.5842	0.7332	**0.8151**	0.4612	0.5952	0.7171	0.8311
	MAPE	0.0536	0.0689	**0.0863**	**0.0946**	0.0542	0.0696	0.0835	0.0967
	$R^2$	0.8958	0.8349	0.7466	0.6805	0.8922	0.8242	0.7509	0.6785
CNN-BiLSTM	MSE	0.4062	0.8011	1.1218	1.4227	0.4033	0.8278	1.1848	1.3846
	MAE	0.4373	0.6526	0.7709	0.8775	0.4449	0.6552	0.7913	0.8653
	MAPE	0.0518	0.0781	0.0910	0.1044	0.0533	0.0774	0.0930	0.1037
	$R^2$	0.9010	0.8035	0.7229	0.6463	0.9017	0.7969	0.7072	0.6559
EEMD-BiLSTM	MSE	0.2557	0.5120	0.8751	1.2395	0.2629	0.5400	0.8999	**1.1808**
	MAE	0.3464	0.4881	0.6699	0.8187	0.3756	0.5071	0.6680	**0.7855**
	MAPE	0.0340	0.0577	0.0823	0.0989	0.0451	0.0610	0.0801	**0.0916**
	$R^2$	0.9377	0.8745	0.7843	0.6925	0.9359	0.8677	0.7783	**0.7072**
Ours	MSE	**0.2306**	**0.4521**	**0.8685**	**1.1987**	**0.2037**	**0.5051**	**0.7978**	1.2857
	MAE	**0.3477**	**0.4921**	**0.6917**	0.8192	**0.3389**	**0.5011**	**0.6216**	0.8150
	MAPE	**0.0417**	**0.0619**	0.0845	0.1021	**0.0406**	**0.0583**	**0.0721**	0.0947
	$R^2$	**0.9438**	**0.8892**	**0.7859**	**0.7028**	**0.9504**	**0.8763**	**0.8034**	0.6809

It is evident that the inclusion of the CNN module enhances prediction performance at step 1. However, its influence diminishes as the step size escalates. Conversely, the introduction of the EEMD decomposition module leads to marked improvements in prediction performance, attaining the second-best results consistently across all step sizes for both Combinations 1 and 2. This underscores that EEMD contributes more significantly towards enhancing predictions compared to the CNN module.

## Discussion and conclusion

Given the seasonal, periodic, uncertain, nonlinear, and intricate interdependencies among indicators within water environmental monitoring data, this paper introduces a hybrid CNN-BiLSTM model integrated with EEMD decomposition for water quality data prediction.

The EEMD decomposition technique is highly effective in mitigating noise interference within the data. Additionally, the four resulting modes from this decomposition process augment the data available for model training, thereby enhancing the training efficacy of the model. The incorporation of CNN enables the model to excel in extracting local features, and its integration with BiLSTM facilitates the utilization of bidirectional data and the acquisition of higher-level features, collectively bolstering prediction performance.

Based on prediction experiments conducted on the dissolved oxygen indicator, the proposed model in this paper demonstrates superior prediction performance compared to existing models. This constitutes a valuable exploration of the practical applications of artificial intelligence technology in the realm of water resource protection. In future, the determination of modal quantity in EEMD, data augmentation for water quality data and and the application of Transformers in long-term water quality data prediction would be beneficial research directions.

In conclusion, the proposed hybrid deep learning approach provides an effective solution for precise multi-step water quality forecasting, capable of addressing the intricate attributes of water environment data. The findings underscore the viability of harnessing advanced AI techniques to enhance environmental modeling and conservation efforts.

## Data Availability

The datasets used and analyzed during the current study are available from the corresponding author upon reasonable request.
